# Assessment of Compliance with National and International Guidelines in the Empirical Management of Community-Acquired Pneumonia (CAP) in Lebanese Hospitals: A Multicenter Retrospective Cohort Study

**DOI:** 10.3390/antibiotics15060551

**Published:** 2026-05-30

**Authors:** Ramona Nasr, Elias A. Rahal, Chadia Haddad, Pascale Salameh, Abir Abdel Rahman

**Affiliations:** 1 Doctoral School of Sciences and Technology, Lebanese University, Hadat 1683, Lebanon; ramona.nasr@ul.edu.lb; 2 Department of Liberal Education, Lebanese American University, Beirut 4504, Lebanon; 3 Department of Experimental Pathology, Immunology, and Microbiology, American University of Beirut, Beirut 1107, Lebanon; er00@aub.edu.lb; 4 Center for Infectious Diseases Research (CIDR), American University of Beirut, Beirut 1107, Lebanon; 5 Institut National de Santé Publique, d’Épidémiologie Clinique et de Toxicologie-Liban (INSPECT-LB), Beirut 1100, Lebanon; 6 Research Department, Psychiatric Hospital of the Cross, Jal Eddib 1525, Lebanon; 7 Faculty of Public Health, Lebanese University, Fanar 2611, Lebanon; 8 Faculty of Pharmacy, Lebanese University, Hadat 1103, Lebanon; 9 Department of Primary Care and Population Health, University of Nicosia Medical School, Nicosia 2417, Cyprus; 10 Medical Laboratory Sciences Program, Faculty of Health Sciences, University of Balamand, Beirut 1300, Lebanon

**Keywords:** community-acquired pneumonia, guideline adherence, empirical antibiotic therapy, antimicrobial stewardship, Lebanon

## Abstract

**Background:** Community-acquired pneumonia (CAP) is a major cause of morbidity and mortality globally, with serious implications in Lebanon. Both international and local guidelines advocate for empirical antibiotic treatments by illness severity, yet the extent to which these are followed in Lebanese hospitals is unclear. This research examined the adherence to CAP treatment guidelines and its association with clinical outcomes. **Methods:** We retrospectively studied adults admitted to two Lebanese referral hospitals (Mount Lebanon University Medical Center and Ain Wazein Medical Village) from April 2011 to March 2025 with CAP. Adherence to empirical antibiotic regimens was determined based on the guidelines from the Lebanese Society of Infectious Diseases and Clinical Microbiology, American Thoracic Society/Infectious Diseases Society of America, and British Thoracic Society/National Institute for Health and Care Excellence. The outcomes assessed were in-hospital mortality, Intensive Care Unit (ICU) admission, and length of hospital stay (LOS). We used logistic and linear regression analyses, adjusting for demographic and clinical variables. **Results:** A total of 337 patients were included with an average age of 61 years; 53.7% were male, 51.6% were admitted to the ICU, and the in-hospital mortality rate was 27%. In general, 65.6% of the treatment regimens adhered to at least one guideline. The combination of β-lactam and macrolide was the most common, used in 87.8% of cases, while monotherapy was administered in 31.8% of cases and included β-lactam, macrolide, fluoroquinolone, and other antibiotics; most monotherapies were non-adherent to guidelines, except for selected fluoroquinolone monotherapy cases that may be considered guideline-concordant under ATS/IDSA recommendations depending on clinical context. Adherence to guidelines did not significantly affect mortality rates (25.8% vs. 29.3%), ICU admissions (52.5% vs. 50.0%), or length of stay (11.4 vs. 9.3 days). Multivariate analysis revealed that older age (OR 1.025, 95% CI 1.008–1.042) and ICU admission (OR 1.024, 95% CI 1.012–1.039) were independent predictors of adverse outcomes, whereas guideline adherence, comorbidities, and inflammatory markers were not independently linked. Surprisingly, mortality was higher among younger patients (average age 58 vs. 67 years, *p* < 0.001). **Conclusions:** Although guideline-concordant empirical therapy was prevalent in this two-center Lebanese retrospective population, it did not independently correlate with length of stay following adjustment, ICU admission, or in-hospital mortality. Patient-related and clinical factors, such as the severity of the illness, may have an impact on observed differences in outcomes, which should be taken as relationships.

## 1. Background

Antibiotics use has significantly reduced mortality from bacterial infections [1], but inappropriate use has accelerated antimicrobial resistance [2], an emerging threat in Lebanese hospitals. In acute-care facilities, antibiotics are frequently misused, especially for respiratory infections, which are among the leading causes of community-acquired infections (CAIs) [3]. Although the surveillance in Lebanon is limited, a multicenter study found that, in comparison to susceptible infections, antimicrobial-resistant CAIs resulted in an extra 2.2 days of hospitalization and $889 extra costs [4].

Community-acquired pneumonia (CAP) is one of the most common and severe community-acquired infections (CAIs) worldwide and in Lebanon [5,6]. The incidence differs between populations and geographical areas. High CAP rates occur in children <6 years in developing and newly industrialized country contexts, which are mainly caused by bacterial agents like *Streptococcus pneumoniae*, *Haemophilus influenzae*, and *Mycoplasma pneumoniae* [7]. In older adults and patients with comorbidities, CAP remains an important health threat. High rates of hospitalization, mortality, and length of stay among non-ICU inpatients have been documented, and death is estimated to occur in 10–12% of general inpatients and in as many as 25% of more severe hospitalizations [8]. Although there are few epidemiological studies available, hospital-based data shows a significant disease burden of CAP in Lebanon, especially among older adults and patients with comorbidities with high ICU admission rates and notable in-hospital mortality reported in Lebanese cohorts [6,9].

A major cause of community-acquired pneumonia (CAP), *Streptococcus pneumoniae*, is becoming more difficult to treat empirically due to growing antimicrobial resistance, especially in Middle East and low- and middle-income countries, where diagnostic capacity and microbiological surveillance remain insufficient, driven by empirical prescribing, limited stewardship, and weak laboratory/surveillance capacity, thereby complicating antibiotic selection and increasing the risk of treatment failure [6,10,11]. Current international guidelines recommend a β-lactam-plus-macrolide combination or fluoroquinolone monotherapy for inpatients with CAP, particularly in severe disease or where resistance is high [12,13]. Penicillin-sensitive strains may respond to the usual β-lactams, but resistant pneumococci may necessitate higher doses or alternative agents such as third-generation cephalosporins and antipneumococcal fluoroquinolones [12]. Even though fluoroquinolones are still useful, their use is limited in order to curb further resistance. Pneumococcal vaccination has also resulted in reduced disease burden in children; however, more wide-ranging effects of the vaccines on resistance patterns in the adult population are still under testing [14]. These concerns highlight the importance of antimicrobial stewardship and local surveillance systems to help optimize empirical therapy and to contribute to better CAP outcomes [15,16].

To guide antibiotic use, many guidelines have been developed internationally and nationally. The American Thoracic Society (ATS)/Infectious Diseases Society of America (IDSA) [5], British Thoracic Society (BTS)/National Institute for Health and Care Excellence (NICE) [17], and Lebanese Society of Infectious Diseases and Clinical Microbiology (LSIDCM) [18] all emphasize empirical treatment that is stratified according to disease severity and resistance trends. However, due to the retrospective nature of our dataset and the lack of routinely documented PSI and CURB-65scores, ICU admission was used as a proxy indicator to pragmatically approximate illness severity. The LSIDCM guidelines recommend a β-lactam and macrolide combination for non-ICU inpatients and suggest a reserve of fluoroquinolones. Despite minor variations in recommendations about the length of therapy and the scheduling of reviews, these guidelines are all dedicated to antimicrobial stewardship for specific patients because of resistance [18]. ATS/IDSA guidelines also emphasize individualized care and following local resistance patterns [5,19], while BTS/NICE guidelines emphasize prompt diagnosis and timely initiation of therapy [17]. Increasing resistance to β-lactams and macrolides has complicated empirical selection, but pneumococcal vaccination has helped reduce invasive disease and penicillin resistance [20]. All sets of guidelines endorse major stewardship principles like early de-escalation, appropriate treatment duration, and sa ecure switch from intravenous (IV) to oral, stressing the importance of flexible and context-specific strategies to address CAP [21,22].

Despite the existence of national guidelines in Lebanon, empirical antibiotic practices in Lebanese hospitals have yet to be evaluated thoroughly. This study uses real-world data from two Lebanese referral hospitals to measure compliance with guidelines and assess the impact of adherence or deviation on patient outcomes. The results will inform antibiotic stewardship strategies and support implementation of evidence-based decision-making in Lebanese acute-care settings.

## 2. Results

### 2.1. Patient Characteristics

#### Sample Description

To summarize empirical prescribing patterns in this cohort, we initially describe the distribution of regimens by macrolide inclusion; the main goal of the study is subsequently to offer assessments of guideline adherence. A total of 337 patients were included in the study; 240 (71.2%) received macrolide therapy, whereas 97 (28.8%) did not. More than half of the participants were male (53.7%) and non-smokers (57.6%), and they had a mean age of 61.18 ± 20.04 years. Regarding comorbidities, 50.7% of participants had hypertension, 36.8% had diabetes, and 26.7% had congestive heart failure. More than half of the participants were admitted to the ICU (51.6%), and 27.0% died during hospitalization.

The mean age (63.68 vs. 54.97, *p* < 0.001) and body mass index (BMI) (26.29 vs. 22.91, *p* < 0.001) of participants were higher among those taking macrolides as compared to the non-macrolide group. Among participants taking macrolide, there was a significantly higher proportion of males as compared to the non-macrolide group (57.9% vs. 43.3%, *p* = 0.015). Additionally, a significantly higher proportion of participants in the macrolide group were smokers as compared to the non-macrolide group (48.3% vs. 27.8%, *p* < 0.001).

Regarding comorbidities, hypertension (55.4% vs. 39.2%, *p* = 0.007) and dyslipidemia (29.2% vs. 3.1%, *p* < 0.001) were more frequent in the macrolide group than in the non-macrolide group. No significant differences were observed for other comorbidities.

ICU admission rates did not differ significantly between the macrolide groups. However, in-hospital mortality was significantly lower among patients who received macrolides compared to those who did not (22.5% vs. 38.1%, *p* = 0.003) (Table 1).

### 2.2. Empirical Antibiotic Therapies and Adherences

Table 2 shows 337 empirical antibiotic regimens, of which 107 were monotherapies (31.8%) and 230 were combination treatments (68.2%). Among monotherapies, β-lactam regimens accounted for the majority (66.4%), followed by macrolides (19.6%), fluoroquinolones (10.3%), and other antibiotics (3.7%). Notably, none of these monotherapies was adherent to national, ATS/IDSA, or BTS/NICE guidelines other than fluoroquinolone monotherapy approved by ATS/IDSA according to specific conditions. Overall adherence was defined as concordance with at least one of the referenced guidelines (LSIDCM, ATS/IDSA, or BTS/NICE); where recommendations differed, a regimen was considered adherent if it matched any guideline framework applicable to the patient’s severity category.

Combination therapy was more commonly observed, with β-lactam + macrolide regimens (87.8%) representing the overwhelming majority. All three guidelines consistently followed this approach. On the other hand, the combination of β-lactam and fluoroquinolone (4.8%) was also in line with the guidelines but was less frequently used. Meanwhile, dual or triple therapies like β-lactam with fluoroquinolone and macrolide (3.9%), as well as fluoroquinolone with macrolide (3.5%), did not adhere to the guidelines.

Overall, 65.6% of regimens complied with at least one guideline, with adherence to empirical therapy advised by guidelines being 53.1% according to LSIDCM, 61.4% according to ATS/IDSA, and 57.0% according to BTS/NICE. The majority of treatments were given orally (76.3%), with intravenous use in 23.8%, and for brief periods of one to six days (93.3%).

#### Guideline Adherence and Clinical Outcomes

Table 3 indicates that a comparison between patients receiving guideline-adherent therapy with those on non-adherent regimens revealed non-statistically significant differences in hospital mortality (25.8% vs. 29.3%, *p* = 0.286), length of stay (11.42 ± 8.12 vs. 9.28 ± 8.12 days, *p* = 0.088), and ICU admission (52.5% vs. 50.0%, *p* = 0.375). However, antibiotic exposure, measured in Defined Daily Doses (DDD), was significantly greater in the adherent group (1.39 ± 0.38 vs. 1.12 ± 0.44, *p* < 0.001), probably reflecting the increased usage of combination regimens (e.g., β-lactam plus macrolide) as advised by guidelines rather than the wider antimicrobial spectrum in general, as DDD is a measure of consumption and not a direct measure of spectrum.

### 2.3. Baseline and Clinical Characteristics by Outcome

Table 4 indicates that in-hospital mortality was unexpectedly higher in younger patients (58 vs. 67 years, *p* < 0.001), with a larger percentage of deaths occurring in the 18–64 age group (52.5% vs. 34.1%, *p* = 0.008).

Admission to the ICU was significantly associated with age (65.3 compared to 56.7 years, *p* < 0.001) and higher comorbidity burden (CCI 1.76 versus 1.33, *p* = 0.002). Few comorbidities were more common in those hospitalized in the ICU, including hypertension (*p* = 0.002), COPD (*p* = 0.004), and CHF (*p* = 0.003).

While the duration of stay was generally comparable among the groups, it was extended for patients treated according to ATS/IDSA (*p* = 0.032) and BTS/NICE (*p* = 0.015) guidelines; these differences should be interpreted as observational associations and may be confounded by baseline disease severity (e.g., higher acuity prompting guideline-concordant combination therapy), particularly given the absence of validated severity scores.

### 2.4. Multivariate Analysis

After adjustment for confounders, age was an independent predictor of both in-hospital mortality (OR 1.025 [95% CI 1.008–1.042], *p* = 0.003) and ICU admission (OR 1.024 [95% CI 1.012–1.039], *p* < 0.001) but not of length of stay.

In contrast, guideline-adherent treatment, inflammatory markers (CRP, ESR, and procalcitonin), and comorbidity were independently not associated with mortality, ICU admission, or LOS after adjustment for available covariates.

Overall, the multivariate analysis underscores that older age and ICU admission were strong risk factors for poor outcomes, whereas no significant protective effect was demonstrated for guideline adherence, inflammatory biomarkers, or comorbidity burden (Table 5).

## 3. Discussion

Our patient cohort had a significant burden of disease, with a mean age of 61, more than 50% admitted to an ICU, and 27% in-hospital mortality. These numbers are comparable to previous ones around a mortality of >20–25% in moderate/severe CAP cases, reflecting the acuity of our real-world patients and concordant with global epidemiology in hospitalized pneumonia patients [23].

The most notable finding in our study was the markedly lower mortality rates in patients receiving macrolide treatment (22.5% compared to 38.1%). This result is in line with evidence reported in existing international research. In a large cohort from Barcelona, where microbial causes were identified, BL–M showed a survival benefit for patients with pneumococcal CAP who exhibited elevated inflammatory markers (adjusted OR 0.28) [24]. Similarly, other cohort studies and larger meta-analyses indicate that BL–M regimens lead to a reduction in mortality compared to either β-lactam monotherapy or fluoroquinolones [25,26]. It is believed that the advantages of macrolides stem from their anti-inflammatory and immunomodulatory properties, beyond their antimicrobial coverage of atypical pathogens [27,28].

In our study, although β-lactam-plus-macrolide regimens were the predominant and guideline-concordant choice, adherence overall was only about 66%, indicating persistent gaps in empirical prescribing, with one third of patients receiving non-adherent regimens. Our adherence rate far exceeds that reported in some middle-income settings, such as Hungary (30%), and no significant mortality benefit was noted (15.6% vs. 16.7%, *p* > 0.05) [29]. In contrast, in well-resourced settings, adherence to guidelines is frequently associated with improved outcomes; an Italian cohort, for instance, reported reduced in-hospital/30-day mortality and shorter LOS with better adherence [30,31,32].

Interestingly, in our adjusted analysis, guideline adherence was not significantly associated with mortality, ICU admission, or LOS. This is consistent with findings from the Hungary study, which showed that increased adherence did not substantially lower mortality [29]. The results indicate that in many real-world contexts, the outcomes of CAP are frequently influenced more by factors related to the patient, such as their age and the severity of their illness, rather than by strict compliance with guideline-recommended treatments.

In line with international CAP research, our investigation identified age and ICU admission as independent predictors of adverse outcomes. Comorbidities, however, were not significantly associated with outcomes in our multivariate model. This aligns with well-established prediction models like the Pneumonia Severity Index (PSI), which emphasize age and comorbidities as crucial factors influencing the severity and outcomes of CAP [33]. These findings are also consistent with validated severity scores such as the PSI [34] and CURB-65 [35], both of which highlight age and comorbidities as key determinants of prognosis. Unexpectedly, there was more mortality in younger patients than older ones (58 vs. 67 years, *p* < 0.001). The higher mortality observed among younger patients may reflect selection bias toward more severe presentations or fulminant disease in ICU-based cohorts, as previously described [36]. Interpretation of these findings remains limited by the absence of systematically recorded severity scores (PSI/CURB-65), restricting precise risk stratification.

In terms of length of stay, patients managed under ATS/IDSA and BTS/NICE guidelines unexpectedly had extended hospitalizations, even though there were no notable differences in mortality rates or ICU admissions. This association may reflect confounding by severity, as more severe cases are more likely to receive guideline-adherent regimens and to require prolonged hospitalization.

Additionally, we found that in multivariate analysis, inflammatory biomarkers (CRP, ESR, and PCT) did not independently predict outcomes. While some research has indicated that biomarkers, especially CRP, may have a prognostic role in severe CAP, the findings are inconsistent. This inconsistency is likely due to variations in measurement timing, laboratory techniques, and patient characteristics. Consequently, the additional value of these biomarkers in predicting outcomes remains a topic of debate [37,38]. Large European studies have also confirmed that after adjusting for severity scores, CRP and PCT have limited ability to predict outcomes, and their differences between bacterial and viral CAP add further complexity to their interpretation. This was also confirmed in large European cohorts, where CRP and PCT showed limited prognostic power after adjustment for severity scores [39], and their variability across bacterial and viral CAP further complicates interpretation [40]. This implies that although biomarkers can help with clinical evaluation, their incremental prognostic value remains limited.

From a stewardship viewpoint, this research underscores both promising trends and notable gaps. The widespread use of BL–M regimens indicates that clinicians are aware of guideline-concordant first-line treatments. Nevertheless, the continued use of monotherapy and non-adherent regimens containing fluoroquinolones, along with significant antibiotic exposure without clinical advantage, reveals areas for improvement. Randomized evidence has confirmed that short-course therapy (5 days) is non-inferior to longer durations [41], and de-escalation strategies are widely recommended to reduce resistance risk [42]. It is essential to systematically integrate interventions such as de-escalation techniques, early transition from IV to oral medication, and short-duration therapy into practice, as these are endorsed by the international stewardship literature. This approach is especially crucial in Lebanon, where the increase in antimicrobial resistance and limited ICU resources necessitate careful management of antibiotics.

In conclusion, our study confirms that older age and ICU admission were strong independent predictors of poor outcomes, emphasizes the absence of an independent effect of guideline adherence, comorbidity, or inflammatory biomarkers on mortality, ICU admission, or length of stay, and identifies key opportunities for stewardship interventions specifically adapted to the Lebanese setting. These findings should be interpreted with caution, as the absence of validated severity scores (e.g., PSI or CURB-65) may result in residual confounding by disease severity despite multivariable adjustment.

## 4. Methods

Between April 2011 and March 2025, we conducted a multicentre retrospective cohort study to assess adherence to national and international guidelines in the empirical treatment of community-acquired pneumonia (CAP) and its impact on clinical outcomes in Lebanon. Data were collected from two tertiary referral hospitals: Mount Lebanon University Medical Center (440 beds, Beirut) and Ain Wazein Medical Village (286 beds, Mount Lebanon), recruiting from different geographical parts of the country, hence increasing the representativeness of the study to the national healthcare landscape. Patient data were retrieved for research at Ain Wazein Medical Village on 14 January 2025 and at Mount Lebanon University Medical Center on 1 April 2025. For each potentially eligible admission, trained clinicians screened the admission files, laboratory reports, and electronic medical records (EMRs) and then reviewed the full chart to confirm that CAP criteria were met. For confirmed cases, we abstracted demographics, comorbidities, clinical presentation, empirical antibiotic therapy, inflammatory markers, and outcomes using a standardized data collection form. The study design was approved by the respective hospital ethical committees (MLH–ID-2025-002/CRU 404); all methods were performed in accordance with the Declaration of Helsinki [43]. Informed consent for the use of medical records was obtained in compliance with national and international policies. Consent procedures applied to eligible hospitalized CAP cases whose records were included in the final analytical cohort (and/or their legal guardians, when applicable). Confidentiality of patient identities was strictly maintained in compliance with both the Helsinki Declaration [20] and Lebanese data protection regulations.

### 4.1. Patient Selection Criteria

The inclusion criteria for hospitalized patients with CAP/severe CAP were based on the latest Infectious Diseases Society of America/American Thoracic Society (IDSA/ATS) 2019 Guidelines for the management of Community-Acquired Pneumonia [5]. Criteria were as follows: (1) aged 18 years or older, (2) community-acquired infection or onset of symptoms ≤ 48 h after hospital admission, (3) respiratory symptoms (cough, fever, sputum production, or dyspnea, chest pain or change in mental status), (4) radiologic (X-rays of the chest or CT-scan) evidence of pneumonia, (5) meeting the diagnostic criteria for CAP, with cases categorized as severe CAP when severe features were present, and (6) undergoing microbiological evaluation, including blood cultures, sputum cultures, or urinary antigen tests, as part of diagnostic examination. Severity was pragmatically operationalized using ICU admission and other accessible clinical indicators because PSI and CURB-65 scores were not routinely recorded in the medical records.

Patients were eligible if they had full hospitalization records, including recorded empirical antibiotic regimen, comorbidities, length of stay, ICU admission, in-hospital mortality, and inflammatory biomarkers such as C-Reactive Protein (CRP), Erythrocyte Sedimentation Rate (ESR), and procalcitonin (PCT). This enabled assessment of compliance with empirical antibiotic recommendations, as well as the effect of compliance with these recommendations on clinical outcomes.

**Figure 1:**
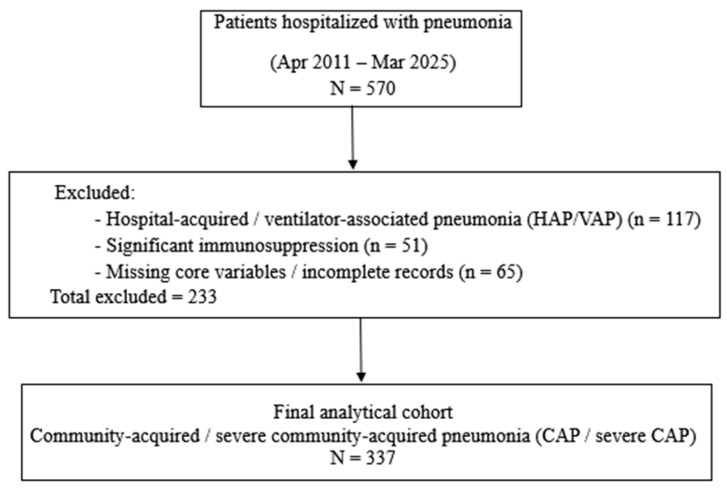
Study Flow Chart.

Exclusion criteria included (1) hospital-acquired pneumonia (HAP) or ventilation-associated pneumonia (VAP), which are associated with different diagnostic and therapeutic strategies; (2) patients with pertinent immunosuppression (i.e., patients with HIV/AIDS, undergoing active chemotherapy, long-term use of immunosuppressant therapy); and (3) missing or incomplete key clinical information in medical records (Figure 1).

Figure 1 summarizes the assembly of the study cohort. Of the 570 adult pneumonia admissions identified across both hospitals during the study period, all 570 had charts retrieved and screened for community-acquired pneumonia (CAP) eligibility. We excluded 117 patients with hospital-acquired or ventilator-associated pneumonia, 51 with major immunosuppression, and 65 admissions with missing core variables required for guideline adherence assessment (empirical regimen, key comorbidities or outcomes). The final analytic cohort comprised 337 unique admissions with community-acquired or severe community-acquired pneumonia (CAP/severe CAP). Where repeat admissions were identifiable in the medical records, they were reviewed during screening to avoid double counting.

### 4.2. Guideline Adherence Assessment

Compliance with empirical antibiotic therapy for CAP was assessed according to national and international clinical practice guidelines, specifically, the Lebanese Society of Infectious Diseases and Clinical Microbiology (LSIDCM 2014) [18], the American Thoracic Society/Infectious Diseases Society of America (ATS/IDSA 2007 and 2019 updates) [5], and the British Thoracic Society/National Institute for Health and Care Excellence (BTS/NICE 2015) [17] [see Supplementary Table S1 for recommended empirical antibiotic therapies for hospitalized patients with CAP]. Empirical antibiotic treatment at each patient’s admission was reviewed and compared with the recommended treatment depending on the severity of the disease, risk factors, and local resistance patterns.

Trained physicians used a standardized data abstraction form to conduct organized chart reviews in order to assess adherence to guidelines. Assessors were not blinded to results due to the retrospective design; nevertheless, in order to reduce subjective interpretation, adherence classification adhered to predetermined criteria based on guidelines. To maintain uniformity, the research team discussed and settled any doubts on regimen classification.

Given the long retrospective study period (April 2011–March 2025), adherence was evaluated by retrospectively benchmarking empirical antibiotic regimens at admission against established national and international CAP guidelines (LSIDCM 2014; BTS/NICE 2015; ATS/IDSA 2007 and 2019 updates), acknowledging that core recommendations for hospitalized CAP; particularly severity-based therapy and β-lactam-plus-macrolide combinations were already present in earlier guidance and remained largely consistent across subsequent revisions. The first empirical antibiotic regimen given at hospital admission (i.e., before microbiological results and before any escalation/de-escalation) was referred to as first therapy, and duration categories (1–6 days vs. ≥7 days) were used to describe short-course versus prolonged therapy patterns in routine practice. Using these designated guideline editions as the reference standard, guideline concordance was assessed consistently for all admissions over the study period instead of allocating distinct guideline editions by calendar year.

Empirical treatment was considered guideline-concordant if both the selected antibiotic and initial dosing scheme were compliant with the specifications presented in the referenced guidelines for the relevant severity class (i.e., non-ICU vs. ICU admission). It was considered as no concordance when there was a deviation in the choice or in the dose of the drug. For combination therapies, all components needed to meet the criteria to be considered adherent. Adherence to dosages was also categorized as appropriate (according with guideline recommendations and if necessary, adjusted for renal function) debatable (slightly incorrect < 50% or loading doses not present) or inappropriate (substantially incorrect ≥ 50%, incorrect frequency, failure to adjust the dose in patients with renal impairment or of extreme body weight [<40 kg or >100 kg]). Only the drug-appropriate regimens were evaluated with respect to dose. The <50% and ≥50% deviation thresholds, which were anchored to the previously mentioned guideline-recommended dosing ranges, were established a priori to differentiate between minor dosing variations that are less likely to significantly alter drug exposure and major dosing errors that may compromise efficacy or increase toxicity (e.g., substantial under/overdosing, incorrect frequency, or failure to adjust for renal impairment) [13,14,15].

The systematic approach allowed for a detailed assessment of real-world prescribing behavior and made possible the categorization of patients according to adherence, with subsequent comparison of patient profiles with clinical outcomes (ICU admission, LOS, in-hospital mortality).

### 4.3. Variables and Outcome Assessment

At baseline, we collected demographics such as age, gender, body mass index (BMI), ideal body weight (IBW), and smoking habits, along with vital signs upon admission, including respiratory rate, blood pressure, oxygen saturation, and confusion status. We also documented comorbidities like allergies, diabetes mellitus, congestive heart failure (CHF), hypertension, chronic obstructive pulmonary disease (COPD), chronic kidney disease (CKD), cancer, rheumatic fever, dyslipidemia, and tuberculosis. The Charlson Comorbidity Index (CCI) was used to assess comorbidities by adding weighted scores for each condition, with higher scores indicating a greater burden. Additionally, we evaluated the severity of comorbidities in organ systems using the Cumulative Illness Rating Scale (CIRS). These conditions were examined as potential factors influencing illness severity and treatment response [44]. For the CCI, comorbidities were identified using ICD-10-CM diagnosis codes from the hospital’s electronic health records and discharge summaries, including those listed on the discharge sheet of the index admission. This involved a 12-month retrospective review, encompassing the index hospitalization, to identify pre-existing conditions. The mapping of ICD-10-CM codes to CCI categories was performed using the validated Quan algorithms [45]. Scores were calculated in R (v4.x) with the comorbidity package [46], utilizing the ICD-10 mapping, and the results were cross-checked against problem lists and discharge diagnoses to ensure comprehensiveness. Drug-resistant infections, such as multi-drug-resistant (MDR) organisms, were also noted when applicable, as they are considered complicating factors in several cases [47]. In order to prevent significant sample size loss and potential bias, PSI and CURB-65 scores were not available, and a number of bedside severity proxies (such as respiratory rate, blood pressure, oxygen saturation, and confusion) were inconsistently recorded over the lengthy retrospective period. As a result, these variables were not included as primary covariates in multivariable models; residual confounding by severity is therefore possible.

The primary outcome was the adherence of empirical antibiotic therapy to national (LSIDCM) and international (ATS/IDSA, BTS/NICE) guidelines in hospitalized adults with CAP, assessed by the choice of the initial empirical regimen. Secondary outcomes encompassed clinical endpoints like mortality during hospitalization, admission to the ICU, and the duration of hospital stay (LOS). Additionally, antibiotic exposure was assessed using Defined Daily Doses (DDD) per patient, and the effect of macrolide therapy on clinical outcomes was evaluated. In order to compare antibiotic use between regimens in this retrospective dataset, DDD was employed as a standardized measure. DDD should be viewed as a consumption metric, nevertheless, as it does not accurately represent the length of therapy or the antibacterial spectrum. Calculating length of therapy (LOT) was not possible since precise treatment days were not consistently recorded across the lengthy trial period. Exploratory analyses also investigated the relationships between initial characteristics, such as age and comorbidities, and patient outcomes. We also classified empirical regimens by macrolide inclusion to describe real-world prescribing patterns, since the β-lactam–macrolide combination is the most common guideline-recommended inpatient regimen. Instead of being used as a stand-alone primary analytical framework, this exploratory and descriptive comparison pertaining to macrolides is offered to contextualize the distribution of guideline-concordant regimens.

### 4.4. Sample Size Calculation

Epi Info version (7.2.5.0) software [48] was used to calculate the necessary sample size. The minimal sample size needed was 231 individuals, assuming an expected frequency of 18.4% for the major outcome (in-hospital death) based on a previous similar study [25], with a 95% confidence level, a margin of error of 5%, and a design effect of 1. To account for potential missing data or exclusions, we increased the sample size by approximately 20%, resulting in a needed sample of 277. We have recruited 337 participants. This sample size was considered sufficient to permit exploratory descriptive comparisons across major empirical regimen groups (such as macrolide-containing vs. non-macrolide regimens) and to address the primary aim (guideline adherence and associated outcomes).

### 4.5. Missing Data and Potential Sources of Bias

For the primary analyses, we required complete information on empirical antibiotic regimen, ICU admission, in-hospital mortality and length of stay. Admissions missing any of these core variables were excluded from the analytic cohort and are reported in the flow diagram (Figure 1). For other variables (for example individual comorbidities or inflammatory markers), we used complete-case analysis and report the number of patients with available data in each table. We did not perform multiple imputation because the proportion of missingness for most covariates was modest and patterns of missing data were not clearly random.

Several sources of potential bias remain. First, the study is restricted to two large referral centers and may not capture CAP management in smaller or public hospitals. Second, excluding admissions with incomplete records may bias the sample towards patients with better documentation or longer hospital stays. Third, misclassification of CAP versus other lower respiratory infections is possible despite systematic chart review. These limitations are considered when interpreting the association between guideline adherence and outcomes.

## 5. Limitations

This research has a number of limitations that need to be recognized. Firstly, its retrospective nature inherently involves the potential for selection bias and unaccounted confounding factors. The quality of the data was dependent on documentation because we relied on hospital records, which could have resulted in missing or insufficient variables. The lack of validated CAP severity scores (PSI and CURB-65), which are essential for risk stratification and outcome prediction, is a significant limitation. Their absence, along with inconsistently reported bedside severity proxies, made it difficult to fully account for baseline disease severity and may have introduced residual confounding by indication. Second, adherence was assessed only at the time of antibiotic initiation but did not capture any subsequent changes, such as de-escalation, escalation, or IV-to-oral switch, which are key aspects of antimicrobial stewardship. Third, the limited microbiological confirmation reflects the practical limitations faced by Lebanese hospitals, which in turn hinders our capacity to associate prescribing habits with appropriateness for specific pathogens. Since microbial etiology and resistance patterns can affect both antibiotic selection and clinical response, the lack of comprehensive microbiological confirmation and pathogen identification may have complicated treatment–outcome correlations. In addition, comorbidity data were limited to variables consistently documented in retrospective records; although we used the Charlson Comorbidity Index (CCI) as a composite measure of comorbidity burden, incomplete capture of specific conditions may have reduced our ability to detect independent associations with outcomes. Moreover, the study was restricted to two referral centers, which may not represent practices across Lebanon. Our findings may not be widely applicable due to differences in diagnostic resources, stewardship practices, and resistance patterns across regions. Biomarkers like CRP, ESR, and PCT were not systematically assessed in standardized time points, which may have reduced our ability to detect dynamic associations between inflammatory response and outcomes. Although the sample size is adequate for identifying significant associations, such as the survival advantage of macrolide therapy, it is still relatively small for conducting subgroup analyses, like those based on macrolide type, administration method, or comorbidity categories. To confirm our results, especially the unexpected finding of increased mortality in younger patients, larger studies across multiple centers are necessary. Additionally, the extended study period (2011–2025) spans multiple updates of national and international CAP guidelines; therefore, adherence was assessed through retrospective benchmarking against established recommendations, which may not fully reflect the guideline versions available at the exact time of prescribing. Furthermore, bedside severity proxies were inconsistently reported and validated severity scores (PSI/CURB-65) were not consistently available, which hindered our ability to completely account for baseline disease severity and left room for possible residual confounding by indication.

## 6. Conclusions

In this retrospective cohort study of hospitalized CAP patients in Lebanon, older age and ICU admission emerged as the strongest independent predictors of poor outcomes. Although guideline-concordant regimens were frequently prescribed, overall adherence was only around two thirds did not independently influence mortality, ICU admission, or length of stay. Similarly, inflammatory biomarkers (CRP, ESR, PCT) and comorbidity burden did not independently predict outcomes in our cohort. These findings should be interpreted as observational and potentially influenced by patient-related and clinical factors, including disease severity. Overall, while guideline-based treatments remain essential for standardizing care, our findings suggest that individual patient characteristics ultimately had a greater influence on prognosis in this setting. These results underscore the need for developing stewardship strategies tailored to local needs and conducting larger multicenter studies to confirm our findings, especially regarding the unexpectedly higher mortality rates observed in younger patients.

## Figures and Tables

**Table 1:** Baseline Characteristics of Hospitalized CAP Patients.

Variable	Total (n = 337)	Macrolide Group (n = 240 (71.2%))	Non-Macrolide Group (n = 97 (28.8%))	*p*-Value
Age (mean ± SD)	61.18 ± 20.04	63.68 ± 19.27	54.97 ± 20.55	<0.001
Gender (M/F)				
Male	181 (53.7%)	139 (57.9%)	42 (43.3%)	0.015
Female	156 (46.3%)	101 (42.1%)	55 (56.7%)	
BMI (mean ± SD)	25.21 ± 5.58	26.29 ± 5.05	22.91 ± 5.95	<0.001
Smoking status (%)	143 (42.4%)	116 (48.3%)	27 (27.8%)	<0.001
Common comorbidities				
Hypertension (%)	171 (50.7%)	133 (55.4%)	38 (39.2%)	0.007
Diabetes (%)	124 (36.8%)	90 (37.5%)	34 (35.1%)	0.673
Chronic obstructive pulmonary disease COPD (%)	40 (11.9%)	32 (13.3%)	8 (8.2%)	0.191
Dyslipidemia (%)	73 (21.7%)	70 (29.2%)	3 (3.1%)	<0.001
Rheumatic fever (%)	2 (0.6%)	2 (0.8%)	0 (0%)	1.000
Tuberculosis (%)	2 (0.6%)	2 (0.8%)	0 (0%)	1.000
Congestive heart failure CHF (%)	90 (26.7%)	59 (24.6%)	31 (32.0%)	0.166
Chronic kidney Disease CKD (%)	23 (6.8%)	16 (6.7%)	7 (7.2%)	0.856
Cander (%)	75 (22.3%)	50 (20.8%)	25 (25.8%)	0.324
ICU admission (%)	174 (51.6%)	121 (50.4%)	53 (54.6%)	0.483
Mortality (%)	91 (27.0%)	54 (22.5%)	37 (38.1%)	0.003

**Table 2:** Empirical Antibiotic Therapies Used.

Antibiotics	Frequency (N)	%	LSIDCM Adherence	ATS/IDSA Adherence	BTS/NICE Adherence
Monotherapy (N = 107; 31.8%)					
β-lactam monotherapy	71	66.4	X	X	X
Fluoroquinolone	11	10.3	X	✓	X
Macrolide	21	19.6	X	X	X
Other antibiotics	4	3.7	X	X	X
Combination therapies (N = 230; 68.2%)					
β-lactam + fluoroquinolone	11	4.8	✓	✓	✓
β-lactam + macrolide	202	87.8	✓	✓	✓
β-lactam + fluoroquinolone + macrolide	9	3.9	X	X	X
Fluoroquinolone + macrolide	8	3.5	X	X	X
Overall guideline adherence					
Adherence to LSIDCM	179	53.1	-	-	-
Adherence to ATS/IDSA	207	61.4	-	-	-
Adherence to BTS/NICE	192	57	-	-	-
Adherence to at least one guideline	221	65.6	-	-	-
Route of administration of the first therapy					
Oral	183	76.3	-	-	-
IV	57	23.8	-	-	-
Duration of total antibiotic therapies					
Long course (≥7 days)	13	6.7	-	-	-
Short course (1–6 days)	223	93.3	-	-	-

X: Non-Adherent; ✓: Adherent.

**Table 3:** Guideline Adherence and Clinical Outcomes.

Variable	Adherent (n=)	Non-Adherent (n=)	*p*-Value
Length of stay (days, mean ± SD)	11.42 ± 8.12	9.28 ± 8.12	0.088
ICU admission (%)	116 (52.5%)	58 (50%)	0.375
In-hospital mortality (%)	57 (25.8%)	34 (29.3%)	0.286
DDD (Defined Daily Doses)	1.39 ± 0.38	1.12 ± 0.44	<0.001

**Table 4:** Comparison of baseline and clinical characteristics in in-hospital mortality, length of hospital stay, and ICU admission in patients with CAP.

		In Hospital Mortality			Length of Hospital Stay		ICU Admission		
		Non-Survivors	Survivors	*p*-Value	Mean ± SD	*p*-Value	Yes	No	*p*-Value
Age	Mean ± SD	58 ± 21.22	67.38 ± 15.34	<0.001	r = 0.115	0.073	65.30 ± 17.78	56.65 ± 21.59	<0.001
Gender	Male	135 (54.9%)	46 (50.5%)	0.279	10.18 ± 9.21	0.699	96 (55.2%)	85 (52.1%)	
	Female	111 (45.1%)	45 (49.5%)		10.68 ± 10.65		78 (44.8%)	78 (47.9%)	0.327
Smoking status (%)	Yes	111 (45.1%)	32 (35.2%)	0.064	9.99 ± 8.93	0.606	73 (42%)	93 (57.1%)	0.471
	No	135 (54.9%)	59 (64.8%)		10.65 ± 10.42		101 (58%)	70 (42.9%)	
CCI Score (comorbidity)	Mean ± SD	1.49 ± 1.28	1.74 ± 1.24	0.109	R = 0.095	0.139	1.76 ± 1.28	1.33 ± 1.22	0.002
Hypertension (%)	Yes	120 (48.8%)	51 (56%)	0.144	10.77 ± 10.36	0.624	102 (58.6%)	69 (42.3%)	
	No	126 (51.2%)	40 (44%)		10.13 ± 9.58		72 (41.4%)	94 (57.7%)	0.002
Diabetes (%)	Yes	87 (35.4%)	37 (40.7%)	0.221	10.3 ± 10.82		70 (40.2%)	54 (33.1%)	
	No	159 (64.6%)	54 (59.3%)		10.67 ± 7.93	0.761	104 (59.8%)	109 (66.9%)	0.108
COPD (%)	Yes	27 (11%)	13 (14.3%)	0.256	13.56 ± 11.51	0.138	29 (16.7%)	11 (6.7%)	
	No	219 (89%)	78 (85.7%)		10.03 ± 9.66		145 (83.3%)	152 (93.3%)	0.004
CHF (%)	Yes	61 (24.8%)	29 (31.9%)	0.123	11.62 ± 9.64	0.313	58 (33.3%)	32 (19.6%)	
	No	185 (75.2%)	62 (68.1%)		10.09 ± 10.003		116 (66.7%)	131 (80.4%)	0.003
Adherence to guidelines									
Adherence to LSIDCM	Yes	133 (54.1%)	46 (50.5%)	0.326	11.39 ± 11.002	0.155	90 (517%)	89 (54.6%)	
	No	113 (45.9%)	45 (49.5%)		9.55 ± 8.80		84 (48.3%)	74 (45.4%)	0.337
Adherence to ATS/IDSA	Yes	143 (58.1%)	49 (53.8%)		11.82 ± 11.57	0.032	94 (54%)	98 (60.1%)	
	No	103 (41.9%)	42 (46.2%)	0.280	9.08 ± 7.84		80 (46%)	65 (39.9%)	0.154
Adherence to BTS/NICE	Yes	155 (63%)	52 (57.1%)	0.196	12.08 ± 11.65		115 (66.1%)	92 (56.4%)	
	No	91 (37%)	39 (42.9%)		8.91 ± 7.77	0.015	59 (33.9%)	71 (43.6%)	0.044
Inflammatory markers									
CRP (mg/L) at admission		16.09 ± 17.19	14.21 ± 14.44	0.320	r = 0.027	0.685	14.3 ± 13.85	16.97 ± 18.92	0.149
ESR (mm/h) at admission		29.70 ± 18.82	27.53 ± 16.98	0.358	r = 0.074	0.272	28.61 ± 17.86	29.68 ± 18.90	0.609
Procalcitonin (ng/mL) at admissions		3.80 ± 7.01	3.43 ± 8.13	0.719	r = 0.074	0.272	3.28 ± 7.13	4.16 ± 7.54	0.313

**Table 5:** Multivariate Logistic Regression Analysis.

Variable	Outcome: In-Hospital Mortality (OR, 95% CI)	*p*-Value	Outcome: ICU Admission (OR, 95% CI, *p*)	*p*-Value	LOS (Beta, 95% CI, *p*)	*p*-Value
Age per 1-year increase	1.025 (1.008, 1.042)	0.003	1.024 (1.012, 1.039)	<0.001	0.066 (−0.039, 0.105)	0.365
Guideline-adherent therapy	1.603 (0.679, 3.787)	0.282	1.066 (0.545–2.084)	0.852	0.083 (−1.74, 5.18)	0.329
CRP level at admission	1 (0.980, 1.020)	0.994	0.989 (0.973, 1.006)	0.218	0.056 (−0.067, 0.162)	0.419
ESR level at admission	0.996 (0.980, 1.012)	0.636	0.999 (0.989, 1.013)	0.938	0.074 (−0.036, 0.118)	0.291
Procalcitonin level at admission	0.998 (0.961–1.036)	0.915	0.981 (0.948, 1.015)	0.267	0.008 (−0.241, 0.272)	0.905
Comorbidities (e.g., CHF, CKD) (0–1 vs. >1 comorbidities)	0.964 (0.522, 1.782)	0.907	1.344 (0.784, 2.30)	0.283	−0.003 (−3.081, 2.935)	0.962

## Data Availability

All data generated or analyzed during this study are included in this published article (and its Supplementary File).

## Supplementary Materials

The following supporting information can be downloaded at https://www.mdpi.com/article/10.3390/antibiotics15060551/s1. Table S1. Recommended Empirical Antibiotic Therapies for Hospitalized Patients with CAP.

## Abbreviations

CAP	Community-Acquired Pneumonia
LSIDCM	Lebanese Society of Infectious Diseases and Clinical Microbiology
ATS	American Thoracic Society
IDSA	Infectious Diseases Society of America
BTS	British Thoracic Society
NICE	National Institute for Health and Care Excellence
ICU	Intensive Care Unit
LOS	Length of Hospital Stay
OR	Odds Ratio
CI	Confidence Interval
CAIs	Community Acquired Infections
IV	Intravenous
EMDRs	Electronic Medical Records
CRP	C-Reactive Protein
ESR	Erythrocyte Sedimentation Rate
PCT	Procalcitonin
HAP	Hospital-Acquired Pneumonia
VAP	Ventilation-Associated Pneumonia
HIV	Human Immunodeficiency Viruses
AIDS	Acquired Immunodeficiency Syndrome
BMI	Body Mass Index
IBW	Ideal Body Weight
CHF	Congestive Heart Failure
COPD	Chronic Obstructive Pulmonary Disease
CKD	Chronic Kidney Disease
CCI	Charlson Comorbidity Index
CIRS	Cumulative Illness Rating Scale
ICD	International Classification of Disease
MDR	Multi-Drug-Resistant
DDD	Defined Daily Doses
BL–M	β-lactam combined with Macrolide
PSI	Pneumonia Severity Index
