# Supplementary material for: Assessment of Compliance with National and International Guidelines in the Empirical Management of Community-Acquired Pneumonia (CAP) in Lebanese Hospitals: A Multicenter Retrospective Cohort Study
Source: Antibiotics (Basel). 2026 May 30;15(6):551. doi: 10.3390/antibiotics15060551 (PMC13295471; doi:10.3390/antibiotics15060551)
Supplement: Supplementary file 1 [file antibiotics-15-00551-s001.zip › antibiotics-4114743-supplementary.pdf]

Table S1. Recommended Empirical Antibiotic Therapies for Hospitalized Patients with CAP

Clinical Setting	ATS/IDSA (2019)	BTS/NICE (2015)	LSIDCM (2014, Lebanon)
**Inpatient – Non-ICU (moderate)**	Beta-lactam (e.g., ceftriaxone) + **Macrolide** (azithromycin or clarithromycin); or Respiratory Fluoroquinolone	Beta-lactam + **Macrolide**	Beta-lactam (e.g., ceftriaxone) + **Azithromycin**
**Inpatient – Severe/ICU**	Beta-lactam + **Azithromycin** (preferred); or Beta-lactam + **Respiratory Fluoroquinolone**	Broad-spectrum Beta-lactam + **Macrolide or Fluoroquinolone**	Antipseudomonal Beta-lactam + **Azithromycin or Levofloxacin**
**Duration of Treatment**	Minimum 5 days; based on clinical stability	Typically, 5–7 days	5–7 days with de-escalation based on response
